# Potential mechanisms of effects of serum‐derived bovine immunoglobulin/protein isolate therapy in patients with diarrhea‐predominant irritable bowel syndrome

**DOI:** 10.14814/phy2.13170

**Published:** 2017-03-08

**Authors:** Nelson Valentin, Michael Camilleri, Paula Carlson, Sean C. Harrington, Deborah Eckert, Jessica O'Neill, Duane Burton, Jun Chen, Audrey L. Shaw, Andres Acosta

**Affiliations:** ^1^ Clinical Enteric Neuroscience Translational and Epidemiological Research (CENTER)Mayo ClinicRochesterMinnesota; ^2^ Entera Health Inc.CaryNorth Carolina

**Keywords:** Bile acids, gene expression, permeability, tryptophan

## Abstract

Serum‐derived bovine immunoglobulin/protein isolate (SBI), an oral nutritional therapy, is efficacious in diverse diarrheal diseases. In an open‐label study in 15 patients with irritable bowel syndrome‐diarrhea (IBS‐D), we evaluated effects of SBI (5.0 g, twice a day) for 8 weeks on safety, on bowel function and abdominal pain, tryptophan metabolism (K:T ratio), intestinal permeability (^13^C‐mannitol and lactulose excretion), bile acid synthesis (fasting serum FGF‐19 and C4), duodenal and stool microbiome, and the expression of 90 genes related to inflammation, immune function, and tight junctions in duodenal mucosa. Statistical analysis (paired tests, baseline vs. treatment) was based on intention to treat (ITT) principles. One of 15 Caucasian patients (13F, 2M, age 40.3 ± 2.3y, BMI 34.3 ± 3.0 kg/m^2^) withdrew without completing studies. There were improvements in stools/day (decrease, *P* < 0.001), ease of passage (*P* = 0.035), and evacuation (*P* = 0.004) with SBI therapy. Worst pain severity was numerically reduced in last 2 weeks' treatment (*P* = 0.078). Duodenal mucosal mRNA expression; serum C4, FGF‐19, and KT ratio; small bowel or colon permeability; and stool microbiome were not significantly different after SBI therapy, compared to baseline. In duodenal brushings, there was considerable microbiota structure difference (*β* diversity analysis *P* = 0.072, UniFrac) and, on taxonomic analysis, increased abundance of *Proteobacteria Burkholderiales, Firmicutes Catonella,* and unclassified genus organisms with SBI therapy. Thus, SBI therapy for 8 weeks in IBS‐D patients is associated with improved bowel function; the mechanism of benefit is unclear, though there were microbiota structure differences in duodenal brushings. Further studies in patients with low‐grade inflammation and intestinal barrier dysfunction at baseline are indicated.

## Introduction

Irritable bowel syndrome (IBS) is a chronic, relapsing, and remitting gastrointestinal (GI) disorder characterized by recurrent abdominal pain or discomfort associated with altered bowel habits that impair the patients' quality of life (Longstreth et al. 2006). Although the pathogenesis of IBS remains incompletely understood, a variety of factors have been associated, and include: genetic susceptibility (Camilleri and Katzka 2012), infections and other environmental exposures, deficiencies in tight junction proteins, intestinal abnormalities with bile acid metabolism, changes in GI motility, visceral hypersensitivity, dysregulation of the interaction between central and enteric nervous system, and psychosocial factors (Camilleri 2012). Recent studies have highlighted the potential role of low‐grade inflammation (Ohman and Simrén 2010), often in association with alterations in the microbiota composition or metabolism (Jeffery et al. 2012), which may cause changes in the epithelial mucosa leading to increased permeability and subsequent dysregulation of intestinal motility or malabsorption of water, electrolytes, and nutrients (Simrén et al. 2013). This increase in GI permeability may also increase antigenic exposure with immune activation, leading to further inflammation and GI symptoms. Tryptophan metabolism is involved in the synthesis of serotonin and affects T‐cell and microbial functions; these are also putative mechanisms in IBS.

The importance of changes in mucosal function in IBS‐D is supported by research showing altered mRNA expression of pivotal mechanisms in patients with IBS‐D. For example, differences in jejunal mucosal expression (at gene and protein levels) and in the distribution of apical junction complex proteins (Martínez et al. 2012, 2013), as well as reduced zonulin‐1 (ZO‐1) expression in HLA DQ2/8 positive patients with nonceliac IBS‐D (Vazquez‐Roque et al. 2012), are thought to produce alterations in barrier function seen in colonic or jejunal mucosa in patients with IBS‐D.

In addition, we have shown that, relative to healthy controls, patients with IBS‐D had altered mRNA expression in colonic mucosa of factors that could be mapped to a biologically relevant pathway in IBS‐D, based on *P*‐values with correction for false detection rate (FDR). These promising observations require replication. The pathways include changes in gene expression for neurotransmitters (P2RY4 and VIP), ion channels (GUCA2B and PDZD3), cytokines and complement (C4BP4 and CCL20), immune function and stress‐related proteins: TNFSF15, IFIT3, HSPA1A, and HSPA1B); mucosal repair, and cell adhesion (TFF, RBP2, and FN1) (Camilleri et al. 2015b). In addition, with FDR correction, the following genes were significantly upregulated (*q* < 0.05) in small intestinal biopsies taken at baseline (prior to SBI treatment in the study reported here) from the participants with IBS‐D, relative to histologically normal control biopsies: INADL, MAGI1, PPP2R5C, MAPKAPK5, TLR3, and IL‐15 (Camilleri et al. 2016a).

A number of studies have suggested a potential role for serum‐derived bovine immunoglobulin/protein isolate (SBI) as a potential therapy for IBS‐D. The potential mechanisms selected were based on several prior observations that provide the rationale for this study:

1. Support of GI mucosal barrier function with SBI, which has been inferred in previous studies that examined anti‐inflammatory effects or immune reconstitution in response to SBI therapy in diverse conditions such as HIV enteropathy, celiac disease, and weight loss associated with cancer (Asmuth et al. 2013; Jatoi 2013; Wilson et al. 2013).
2. Earlier work in enteropathic or inflammatory models in large non‐human animals (using spray‐dried plasma enriched for immunoglobulin content) showed oral administration resulted in improved weight gain (Torrallardona 2010), as well as gut barrier function and permeability (Moretó and Pérez‐Bosque 2009; Campbell et al. 2010), and a significant reduction in the severity of enteropathy in animals (Kuchibhatla et al. 2015; Perez‐Bosque et al. 2015). However, these studies in animals require replication in humans.
3. More recent clinical studies on SBI have demonstrated that the product is safe and improves nutritional status and gastrointestinal symptoms (e.g., chronic loose and frequent stools, abdominal discomfort, bloating, and urgency) in patients with enteropathy associated with IBS‐D or HIV infection (Asmuth et al. 2013; Wilson et al. 2013). Wilson et al. (2013) showed, in a pilot comparator (10 g/day soy protein isolate) controlled trial of 6 weeks duration, that either 10 g/day or 5 g/day of SBI in 30 patients resulted in statistically significant within‐group improvements in daily symptom scores (e.g., urgency, flatulence, and abdominal pain) in subjects with IBS‐D, though there was no significant benefit over the comparator in the pilot study. Hence, further understanding of the clinical benefits with SBI therapy is required, while exploring the putative mechanisms of action of SBI, the goal of this study.
4. In patients with IBS‐D following recurrent *C.difficile* infection, SBI restored normal bowel function in two patients treated in open‐label fashion (Crawford and Panas, 2015), suggesting potential effects on barrier function, mucosal immune function or, conceivably, the microbiome, but clearly requiring more rigorous study.
5. In a single‐center, retrospective chart review of 45 patients with inflammatory bowel disease (38 Crohn's disease and 7 ulcerative colitis)) with limited to no response to traditional pharmaceutical therapies, the medical food, SBI, has been shown to improve symptom scores and clinical management (Shafran et al. 2015).
6. SBI has been shown to bind and neutralize microbial components (Tomita et al. 1995; Navarro et al. 2007; Detzel et al. 2015).

However, the mechanisms resulting in these improvements in bowel function in IBS‐D are unclear, even though open‐label treatment in case series suggests there is benefit when SBI is added to the patient's current standard care in a clinical practice (Good et al. 2015).

Our aim was to evaluate the potential mechanisms of action in an open‐label study of clinical safety and effectiveness of SBI in patients with IBS‐D. The potential mechanisms underlying the activity were also explored, including changes in epithelial barrier function (for which the study power had sufficient power to detect a biologically relevant change), bile acid synthesis, tryptophan metabolism, and mucosal expression of pivotal genes in small bowel mucosa and duodenal and stool microbiota. These other endpoints were appraised to gather information about other potential mechanisms and their coefficients of variation in response to SBI treatment in order to plan future controlled trials.

## Methods

### Ethical approval

The study was approved by Mayo Clinic Institutional Review Board on June 3, 2014 (IRB #14‐002151). Written informed consent was received from participants prior to inclusion in the study. This trial was registered in ClinicalTrials.gov: #NCT02163213.

### Study design

**Figure 1:**
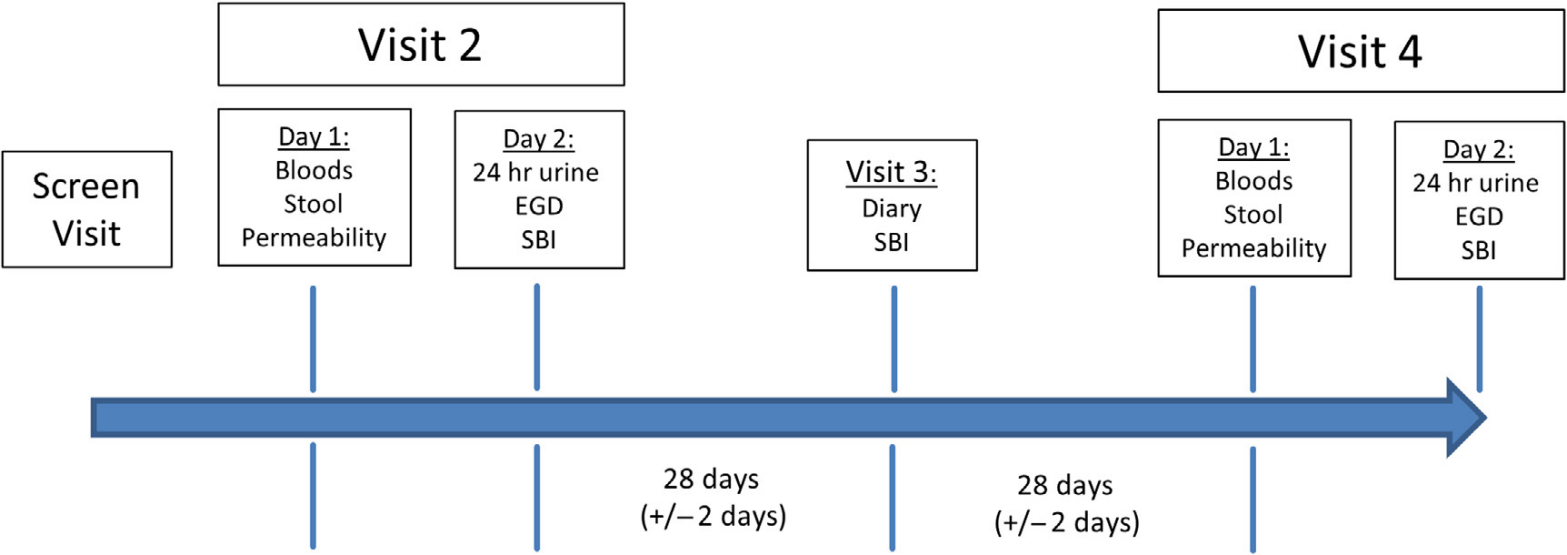
Study Design. After baseline evaluations, participants received SBI treatment for 8 weeks followed by repeat assessment of K:T ratio, bile acid kinetics, mucosal permeability, mRNA expression in duodenal biopsies, and microbiome measurements of duodenal brushings and stool.

In an open‐label study, we evaluated the effects of SBI, 5.0 g (administered as 5‐gram packets), twice daily (BID), for 8 weeks (Fig. 1) in 15 eligible subjects, on GI symptoms, tryptophan metabolism (kynurenine to tryptophan ratio), intestinal permeability (in vivo), microbiota of duodenal brushings, and stool and mucosal expression of pivotal genes, including those for tight junction proteins, secretory mechanisms, tissue repair proteins, and chemokines. Plasma, duodenal, and stool samples were collected. Bowel function and abdominal pain (worst and average) were evaluated using a daily diary including the Bristol Stool Form Scale (BSFS) (Lewis and Heaton 1997). Bile acid homeostasis was assessed by fasting serum FGF‐19 and C4. Intestinal permeability was measured in vivo by two sugar (^13^C‐mannitol and lactulose) urine excretion(s) at 0–2 (small bowel) and 2–8 h (small bowel and colon) after oral ingestion. Biopsies from the distal duodenum were obtained endoscopically to measure mRNA expression of pivotal genes and perform microbiome analysis. Stool samples were collected to perform microbiome analysis. Statistical analysis was based on intent to treat (ITT) principles using paired tests and imputation for missing data.

### Participants

Fifteen male or nonpregnant female patients, aged 18–65 years, with IBS‐D based on Rome III criteria were invited to participate. Patients were randomly selected from a database of approximately 350 patients with IBS‐D who reside within 120 miles of Rochester, MN. Details about eligibility for participation and concomitant medications are provided in ClinicalTrials.gov: #NCT02163213.

For inclusion in the study, each participant's baseline 14‐day bowel diary had to show at least 2 days with >3 BMs/day during each week. Patients were excluded from participation based on intake of medications that might interfere with the study, use of antibiotics within the previous 2 weeks (selected because human fecal microbiome studies show return of fecal microbiome to initial state begins by the end of 1 week and is completely restored by 4 weeks after antibiotic cessation) (Dethlefsen et al. 2008; Dethlefsen and Relman 2011), prior abdominal surgery [except pelvic surgeries, cholecystectomy (as long as symptoms of IBS and diarrhea did not begin or worsen after the surgery), and appendectomy], active gastrointestinal diagnosis other than IBS, history of allergy or intolerance to beef or to any ingredient in SBI, uncontrolled psychiatric disorders (including significant depression or suicidal ideation), intake of NSAIDs or aspirin within the week prior to tests of intestinal permeability (since they all may affect intestinal permeability), bleeding disorders or medications that increase risk of bleeding from mucosal biopsies, and ingestion of artificial sweeteners such as sucralose, aspartame, and foods containing lactulose or mannitol within 2 days of the intestinal permeability test and throughout the 24‐h test period.

### Concomitant therapies

#### Permitted therapy

All concomitant medications (prescriptions and over the counter) used by the subject 30 days prior to signing the informed consent form and during the 8‐week therapy period were recorded. Patients were permitted to continue estrogen replacement, birth control pill, thyroid replacement therapy, and low‐dose tricyclic antidepressants (maximum 50 mg per day).

#### Prohibited therapy

Prohibited therapy included antidiarrhea medications inclusive of opiate‐based medications, bismuth preparations, laxatives or stool softeners, prokinetic agents, fiber supplements (exception: subjects who were on stable treatment with a daily fiber supplementation), probiotics, prebiotics, nutritional supplements (glutamine, zinc, calcium), immunoglobulin supplements, enemas, iron supplements, octreotide, rifaximin, and antibiotics (unless deemed necessary by the investigator for treatment of infection).

### Assessment of stool frequency, consistency, and ease of passage

During the 14‐day baseline (postscreening examination and consent completion) and the 56‐day study period (±4 days), patients completed a daily bowel function diary, which included the 7‐point Bristol Stool Form Scale (BSFS), ranging from 1 or hard lumpy stool to 7 or watery diarrhea (Lewis and Heaton 1997), the timing of each bowel movement, the ease of passage (to assess evacuation) and completeness of evacuation, and any medications taken by the patient. The bowel pattern diary was dispensed at the screening visit, and the completed bowel diary was collected at the end of each week to ensure compliance to regimen.

### Serum markers of bile acid synthesis

Given that a significant portion of patients with IBS‐D has also been reported to have alterations in bile acid homeostasis (Valentin et al. 2016), we evaluated changes in bile acid parameters before and after treatment with SBI therapy for 8 weeks. The two serum markers were fasting 7*α*‐hydroxy‐4‐cholesten‐3‐one (measured by chromatography method) (Camilleri et al. 2009) and fibroblast growth factor‐19 (measured by a commercial FGF‐19 Quantikine Enzyme‐Linked Immunosorbent Assay Kit; R & D Systems, Minneapolis, MN).

### Kynurenine to tryptophan ratio

In order to evaluate tryptophan metabolism, which is involved in the effects of serotonin and affects T‐cell and microbial functions, serum kynurenine to tryptophan (K:T) ratio was measured at baseline and at the end of 8 weeks of SBI therapy. Serum samples were stored and then sent in a batch to a reference laboratory for analysis. Liquid chromatography–tandem mass spectrometry was used to assess kynurenine and tryptophan levels as previously described (Huang et al. 2013).

### Upper GI endoscopy and biopsies

Upper GI endoscopies were performed by gastroenterologists (MC, AC). Patients received conscious sedation as required for the procedure to be conducted with comfort. A combination of benzocaine 20% spray to the throat, midazolam and fentanyl was used as in routine clinical practice. A full endoscopy report was created for each procedure and incorporated in the patient's medical record.

A total of eight biopsies were collected. For the purposes of this study, biopsies were used for mRNA expression and brushings from the duodenal mucosa for microbiome analysis.

### Measurement of small intestinal permeability in vivo

As in prior studies (Camilleri et al. 2010; Rao et al. 2011; Grover et al. 2016), lactulose, 1000 mg, and ^13^C‐mannitol, 200 mg, (L7877 and M8429 from Sigma‐Aldrich, St. Louis, MO 63103) were used to determine the urine sugar excretions at different times as markers of small bowel and colonic mucosal permeability after oral ingestion of the sugars in aqueous solution. Over the first 8 h after saccharide ingestion, urine was collected in two pooled batches: one for the first 2 h (which reflects small bowel permeability), and a second pooled sample over the next 6 h (2–8 h after saccharide ingestion, which reflects both small bowel and colonic permeability). The total volume of each collection was measured, and an aliquot from each collection was obtained to estimate the total content of each sugar for the different time intervals. The urine aliquot was stored at −20° Celsius until it was thawed for analysis.

Participants ingested standardized meals during the first 8 h. Specifically, 500 mL water was given 30 min after sugar administration to aid in the collection of urine. A breakfast of egg, toast and water was given after 2 h, and a lunch of chicken, potato and water was offered after 6 h. Water was allowed ad libitum throughout the day.

We estimated cumulative and ratio excretions of the two sugars at 0–2 h and 2–8 h for small bowel mucosal permeability and large bowel permeability, respectively. Urinary saccharide concentrations were measured by high‐performance liquid chromatography‐tandem mass spectrometry. Details of this method were previously described elsewhere; the assay was adapted from the method of Lostia et al. 2008. Small bowel permeability in patients with IBS‐D in this cohort was compared to the permeability of 12 healthy subjects acquired in a contemporaneous study in our laboratory using the same method of measurement (Camilleri et al. 2010). The procedure was performed at baseline and at the end of treatment period.

### Quantitation of tight junction proteins using real‐time PCR

Real‐time PCR was used to quantitate tight junction proteins in small intestine biopsy samples from all 15 IBS‐D patients at baseline and after treatment. Biopsy samples were submerged in RNAlater Solution (Ambion, Austin, TX) and stored at −80°C.

### Gene expression by RT^2^ PCR array

For mRNA expression, RNA was purified from duodenal biopsies using the Qiagen RNAeasy Kit, including on‐column DNAse treatment to remove genomic DNA. RNA quality was assessed on the Agilent Bioanalyzer. The resulting RNA (RIN >7) was reverse transcribed using the RT^2^ First Strand Kit, (Qiagen) and samples were analyzed for expression by a Custom Profiler RT^2^ PCR Array (Qiagen). In our custom profile, we selected 93 genes [as in our prior study of mRNA expression in duodenal mucosa of patients with IBS‐D (Camilleri et al. 2016a)], three housekeeping genes for normalization (ACTB, B2M, GAPDH), and control gene (positive PCR control, PPC) to check for sample quality and reaction quality. These genes are discussed further in the Results section. Data analysis was performed using the RT^2^ Profiler PCR Array software package. This package uses ΔΔ C_T_–based fold change calculations and the Student's t‐test to calculate two‐tail, equal variance p‐values.

### Safety assessments

In accordance with the safety assessment protocol, we collected information related to monitoring of adverse events (AEs), clinical laboratory testing, vital sign measurements, concomitant medications, and physical examinations.

### Microbiome collection, 16S amplicon preparation, sequencing, processing, and analysis

Duodenal brushings (collected in RNAlater) and fecal samples were frozen and stored at −80°C. Tissue brushings were extracted with the MoBio PowerSoil^®^ DNA Isolation Kit (PN12888 Mo Bio Laboratories, Inc., Carlsbad, CA) according to the manufacturer's protocol.

Stool was extracted in the Biospecimens, Accessioning, and Processing (BAP) Core facility on the Rochester campus of the Mayo Clinic using Chemagic DNA Blood Kit (CMG‐741) and Chemagic MSM Instrumentation (PerkinElmer Chemagen Technologie GmbH). Concentrations were determined by Qubit dsDNA HS Assay Kit (PN Q32854 Thermo, Fisher Scientific Inc., Waltham, MA).

A two‐step PCR protocol was used to amplify the V3‐V5 region of the 16S rRNA gene, as described previously (Gohl et al. 2016). The primary amplification primers were developed in collaboration with the University of Minnesota Genomic Center in Minneapolis, MN. They contain both 16S‐specific primers (V3_357F and V5_926R) (Yu and Morrison 2004) and Nextera adapter tails: forward primer.

5′‐TCGTCGGCAGCGTCAGATGTGTATAAGAGACAGCCTACGGGAGGCAGCAG‐3′; reverse primer.

5′‐GTCTCGTGGGCTCGGAGATGTGTATAAGAGACAGCCGTCAATTCMTTTRAGT‐3′. Indices and flow cell adapters were added in a secondary amplification using Nextera index primers i5 and i7. PCR products were normalized and then pooled to equalize concentrations for sequencing using the MiSeq 600 Cycle v3 Kit (Illumina, San Diego, CA) and MCS v2.6.1 at the Molecular Genomic Facility, Mayo Clinic, Rochester, MN.

The raw 16S data were processed by IM‐TORNADO Bioinformatics Pipeline (Jeraldo et al. 2015). Taxonomy was assigned against a Green Genes reference database (v13.5), and operational taxonomic units (OTUs) were assigned using a 97% identity threshold (Caporaso et al. 2010, 2012).

For statistical analysis, *α*‐diversity (Observed OTU number and Shannon Index) and *β*‐diversity (UniFrac distances) measures were calculated, based on the rarefied OTU counts. UniFrac distance refers to a metric used for comparing biological communities, and it incorporates information on the relative relatedness of community members by incorporating phylogenetic distances between observed organisms in the computation. Linear mixed effects model was used to test for association between *α*‐diversity and treatment status. PERMANOVA was used to test for association between treatment status (pre versus posttreatment) and the overall microbiota composition as captured by *β*‐diversity, based on distance matrices (“adonis” function in R package “vegan”). Significance was assessed by 1000 permutations with permutation constrained within the same subject to account for within‐subject correlation. Differential abundance analysis was performed using the Wilcoxon signed‐rank test at phylum, family, and genus levels. False discovery rate (FDR) control, based on the Benjamini–Hochberg procedure, was used to correct for multiple testing. All the statistical analyses were performed in R‐3.0.2 (R Development Core Teams).

### Other statistical considerations

#### Statistical power calculation

The power calculation was based on the planned primary mechanistic endpoint of small intestinal permeability. Based on prior measurements in our laboratory, using unlabeled mannitol urinary excretion during the first 2 h after ingestion as a probe of small intestinal permeability, we estimated that the 15 patient sample would have sufficient power to detect a 44% change in 0–2 h urinary excretion, in accordance with the following table:

**Table:** 

Response	Mean	SD	COV %	Anticipated width of a 95% CI based on a sample size of 15 [using a 2‐sided t statistic (14 df) at *α *= 0.05]
Urine mannitol mg, 0–2 h	29.8	11.9	40	13.1 (% relative to mean = 44%)
Serum C4, ng/mL	32.7	24.8	76	19.3 (% relative to mean = 59.9%)
Serum FGF‐19, pg/mL	120.5	86.8	72	67.5 (% relative to mean = 56.0%)

Such change in effect size of measurement of permeability is in the range (mean change 53%) observed when elemental diet was used to treat Crohn's disease (Teahon et al. 1991). Similarly, we have shown that the bile acid sequestrant, colesevelam, results in a 200% change in fasting serum C4 level (Camilleri et al. 2015a).

#### Statistical comparisons

The *primary endpoints* for the study were change from baseline in:

1. small intestinal permeability (0–2 h mannitol excretion)
2. mucosal expression of tight junction proteins (ZO‐1, occludin, claudin)

The *secondary endpoints* for the study were change from baseline in:

1. stool frequency, stool consistency (using the Bristol stool form scale), ease of stool passage, abdominal pain over the entire 8‐week treatment period and for the final 2 weeks of treatment
2. duodenal brushings microbiome
3. stool microbiome
4. serum levels of 7‐hydroxy‐4‐cholesten‐3‐one (C4; a surrogate for BA synthesis) and fibroblast growth factor (FGF) 19 (an ileal hormone that downregulates BA synthesis)
5. serum tryptophan metabolism (kyneurenine/tryptophan [K/T] ratio)

All analyses used paired comparisons of the single values on treatment compared to baseline, or the mean measured via daily diaries for symptoms related to bowel function or abdominal pain during 2 weeks' baseline, and over 8 weeks or final 2 weeks of treatment.

## Results

### Baseline demographics and screening

A total of fifteen patients diagnosed with IBS‐D (Rome III) participated in this open‐label study. All were Caucasian (13 female, two male; combined mean age 40.3 ± 2.3 years, combined mean BMI 34.3 ± 3.0 kg/m^2^). During the 2‐week screening period, all participants had at least 2 days/week with at least three bowel movements per day.

### Clinical studies

#### Bowel function

A detailed summary of changes in bowel function can be seen in Table 1. The average number of stools per day decreased from 2.35 to 1.79 (delta: −0.56, *P* < 0.001). Average stool form as assessed by the BSFS (1‐hard lumps to 7‐watery) decreased from 5.13 to 4.87 (delta: −0.26, *P* = 0.07). Average ease of passage decreased from 4.70 to 4.45 (delta: −0.25, *P* = 0.035). The proportion of incomplete evacuation decreased as well from 0.37 to 0.30 (delta: −0.07, *P* = 0.004) after 8 weeks of SBI therapy. There were no significant overall effects on pain during the 8 weeks of therapy, though the worst pain severity was numerically reduced in the last 2 weeks of treatment (*P* = 0.078).

**Table 1:** Symptom scores at baseline and during 8 weeks' treatment with SBI

Daily score over 8 weeks (unless otherwise stated); mean (SEM)	Baseline	Treatment	Delta	*P*‐value
# Stools/day	2.35 (0.20)	1.79 (0.16)	−0.56 (0.09)	<0.001
Stool form‐ (BSFS, range 1–7)	5.13 (0.15)	4.87 (0.17)	−0.26 (0.13)	0.070
Stool ease of passage	4.70 (0.09)	4.45 (0.10)	−0.25 (0.11)	0.035
Proportion with incomplete evacuation	0.37 (0.07)	0.303 (0.07)	−0.07 (0.02)	0.004
Average pain severity over 8 weeks	18.88 (4.42)	15.28 (4.31)	−3.597 (3.68)	0.345
Worst pain severity over 8 weeks	24.38 (4.56)	18.95 (4.46)	−5.433 (3.58)	0.152
Average pain severity last 2 weeks	18.88 (4.42)	12.33 (3.21)	−5.324 (3.74)	0.178
Worst pain severity last 2 weeks	24.38 (4.56)	15.90 (3.83)	−6.545 (3.42)	0.078

#### Compliance and adverse events

All patients showed excellent compliance with therapy, as evaluated by count of returned SBI packets. One patient withdrew without completing the study after reporting an adverse event (AE). Overall, 8 (53%) of 15 patients experienced 20 therapy emergent adverse events (TEAE) during the study (Table 2). The events were mild (45%), moderate (45%), or severe (10%) in intensity. The TEAEs reported included headache (two patients); cramping (two patients); nausea (two patients); gas (two patients); and one patient for each, sores on tongue, metallic taste in mouth, back pain, stomach flu, bloating, leaking, sinus infection, abdominal, acid reflux, cold sore, and sick.

**Table 2:** Treatment emergent adverse events during open‐label study

Subject study ID	Description of adverse event	Intensity	Relationship to the investigational product	Duration of event (# days)	Resolved (Yes/No)
005‐1235	Nausea	Mild	Possibly	16 days	Yes
	Sores on side of tongue	Mild	Possibly	16 days	Yes
	Metallic taste in mouth	Mild	Possibly	16 days	Yes
008‐1275	Back pain	Severe	Not	3 days	Yes
012‐1298	Stomach flu	Moderate	Not	2 days	Yes
013‐1545	Intermittent bloating	Mild	Not	55 days	No
	Intermittent gas	Mild	Not	55 days	No
	Intermittent leaking	Mild	Not	55 days	No
	Intermittent cramping	Mild	Not	55 days	No
	Intermittent nausea	Mild	Not	55 days	No
014‐1300	Sinus infection	Mild	Not	7 days	Yes
016‐1553	Headache	Moderate	Not	1 day	Yes
	Headache	Moderate	Not	1 day	Yes
	Headache	Moderate	Not	1 day	Yes
017‐1550	Intermittent cramping	Moderate	Not	1 day	Yes
	Headache	Moderate	Not	1 day	Yes
	Acid reflux	Severe	Not	1 day	Yes
	Gas	Moderate	Not	1 day	Yes
	Cold sore	Moderate	Not	6 days	Yes
021‐1292	Sick	Moderate	Not	5 days	Yes

Intensity was classified as Mild, Moderate, Severe, Serious.

Relationship to the Investigational Product was classified as related, possibly related, not‐related.

The patient who experienced nausea, mouth sores, and a metallic taste in the mouth discontinued from the study without completing the study procedures. Only these events were considered to be possibly related to the product.

### Mechanistic studies

#### Effects of SBI on intestinal permeability

**Figure 2:**
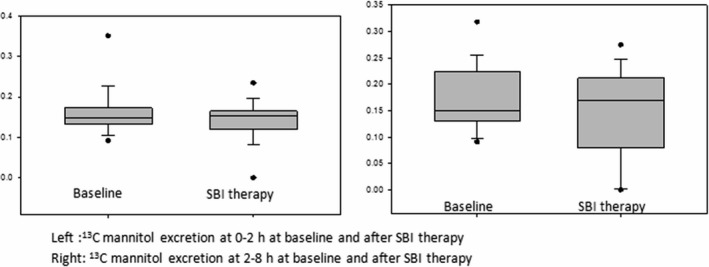
Changes in intestinal permeability from baseline to end of SBI therapy: Note there were no significant changes in ^13^C‐mannitol excretion in the urine during 0–2 h post oral administration of the monosaccharide corresponding to small intestinal permeability. Similarly, the 2–8 h excretion (reflecting predominantly colonic permeability) was not different in the two measurements at baseline and following SBI therapy.

Intestinal permeability, as an indicator of gut barrier function, was assessed by the analysis of the proportion of the original dose of ^13^C‐mannitol excreted in urine over an 8‐h period, as well as the lactulose to mannitol ratio (LMR) of excretion in urine during the same time period. We compared these permeability parameters to those of 12 healthy controls. Excretion of ^13^C‐mannitol between 0 h and 2 h (Fig. 2, left panel) is a marker of small intestinal permeability, while excretion during the 2–8 h period (Fig. 2, right panel) is a marker of both small bowel and colonic permeability. The ^13^C mannitol excretion and the LMR at baseline in the IBS‐D patients did not differ from healthy controls (Table 3). There were no significant changes in small bowel or colonic permeability after SBI therapy for 8 weeks.

**Table 3:** Intestinal permeability, nutritional and bile acid synthesis parameters (serum 7*α* C4 and FGF‐19) at baseline and during 8 weeks' treatment with SBI; data shown are Mean (SEM)

Mean (SEM)	Baseline	Treatment	Delta	*P*‐value
^13^C‐Mannitol excretion, proportion of orally administered dose				
0–2 h	0.162 (0.017)	0.139 (0.014)	0.029 (0.02)	0.177
2–8 h	0.169 (0.017)	0.147 (0.023)	0.027 (0.02)	0.247
Bile acid and nutritional parameters				
Fasting serum FGF‐19, pg/mL	114.6 (16.9)	113.4 (20.3)	−1.14 (11.50)	0.923
Fasting serum 7*α* C4, ng/mL	33.67 (6.9)	31.94 (6.1)	3.35 (3.78)	0.392
Serum kynurenine: tryptophan ratio	0.031 (0.001)	0.032 (0.002)	−0.0007 (0.001)	0.558
Serum kynurenine ng/mL	344.7 (17.2)	335.7 (19.2)	−9.02 (8.59)	0.313
Serum tryptophan, *μ*g/mL	10.95 (0.3)	10.49 (0.40)	−0.463 (0.29)	0.133

Abnormal serum 7*α* C4 > 49.1 ng/mL and abnormal serum FGF‐19 is <79 pg/mL.

We assessed the effect of SBI in six patients who had baseline 0–2 h excretion >0.228 (proportion of administered dose, based on published normal values [Grover et al. 2016]). There was a numerical reduction in excretion (proportion excreted 0.465 ± 0.256 [SD] at baseline compared to 0.301 ± 0.05 on SBI, *P* = 0.173) which was not statistically significant.

#### Tryptophan metabolism

Therapy with SBI for 8 weeks caused no significant change in the kynurenine to tryptophan (K:T) ratio (Table 3). The K:T ratio at baseline and after treatment was 0.031 and 0.032, respectively (*P* = 0.353). Interestingly, there was a borderline reduction in tryptophan from 10.95 *μ*g/mL to 10.49 *μ*g/mL (*P* = 0.133) after SBI therapy.

#### mRNA expression of pivotal genes in duodenal mucosa

To evaluate the impact of SBI therapy on gene expression in the mucosa of the small intestine in patients with IBS‐D, we quantified changes from baseline in the expression of 90 candidate genes (Table 4). All candidate genes, including those encoding tight junction proteins, were related to immunity and inflammation, and represent components of biological pathways involved in the pathogenesis of IBS‐D. The mRNA expression analysis of 90 candidate genes from duodenal mucosa was not significantly different after SBI therapy. There was borderline alteration in fold expression of mRNA of CLDN4 and IL4 genes (1.18, *P* = 0.09; and 0.78, *P* = 0.13, respectively).

**Table 4:** Fold up (+) or down (−) regulation for 90 genes of interest (Q‐values all *P* = NS)

Gene symbol	Official full name	Fold regulation (95% CI)	*P* value
C4BPA	Complement component 4 binding protein, alpha	−1.02 (0.54, 1.42)	0.982
CCL20	Chemokine (C‐C motif) ligand 20	1.49 (0.46, 2.53)	0.246
CLDN1	Claudin 1	−1.06 (0.59, 1.30)	0.835
FGFR4	Fibroblast growth factor receptor 4	1.02 (0.70, 1.34)	0.818
FN1	Fibronectin 1	1.15 (0.76, 1.55)	0.548
GPBAR1	G‐protein‐coupled bile acid receptor 1	−1.08 (0.60, 1.25)	0.976
GUCA2B	Guanylate cyclase activator 2B (uroguanylin)	1.02 (0.66, 1.38)	0.699
IFIT3	Interferon‐induced protein with tetratricopeptide repeats 3	1.06 (0.70, 1.43)	0.484
NR1H4	Nuclear receptor subfamily 1, group H, member 4	1.04 (0.72, 1.36)	0.802
OCLN	Occludin	1.1 (0.83, 1.37)	0.402
P2RY4	Pyrimidinergic receptor P2Y, G‐protein‐coupled, 4	1.36 (0.53, 2.18)	0.626
PDZD3	PDZ domain containing 3	1.11 (0.46, 1.75)	0.530
RBP2	Retinol binding protein 2, cellular	1.03 (0.75, 1.32)	0.901
SLC6A4	5‐HT transporter	1.21 (0.69, 1.74)	0.743
SLC10A2	Solute carrier family 10 member 2 (sodium/bile acid co‐transporter)	−1.04 (0.55, 1.37)	0.613
TFF1	Trefoil factor 1	1 (0.60, 1.40)	0.450
TJP1	Tight junction protein 1 (zona occludens 1)	1.13 (0.85, 1.41)	0.263
TNFSF15	Tumor necrosis factor superfamily, member 15	1.07 (0.65, 1.49)	0.434
VIP	Vasoactive intestinal peptide	1.08 (0.11, 2.05)	0.719
IFNG	Interferon‐gamma	1.04 (0.37, 1.72)	0.922
MYLK	Myosin Light Chain Kinase	1.14 (0.72, 1.56)	0.517
SLC9A1	Na+‐H + ‐exchange protein	1.1 (0.74, 1.46)	0.574
CALR	CALReticulin	1.17 (0.84, 1.50)	0.257
CD3E	CD3 ‐ epsilon chain	1.13 (0.70, 1.56)	0.431
CD74	HLA‐DR antigens‐associated invariant chain	1.14 (0.83, 1.45)	0.541
CLDN12	Claudin 12	1.01 (0.69, 1.33)	0.946
CLDN15	Claudin 15	1.05 (0.86, 1.25)	0.639
CLDN16	Claudin 16	−1 (0.54, 1.45)	0.721
CLDN2	Claudin 2	−1.22 (0.35, 1.28)	0.988
CLDN3	Claudin 3	1.01 (0.81, 1.21)	0.941
CLDN4	Claudin 4	1.18 (0.92, 1.44)	0.089
CLDN7	Claudin 7	1.11 (0.85, 1.36)	0.296
CPSF1	Cleavage and polyadenylation‐specific factor 1	1.04 (0.59, 1.50)	0.787
CTNNA1	Catenin (cadherin‐associated protein), alpha 1	1.08 (0.80, 1.37)	0.444
CTNNB1	Catenin (cadherin‐asociated protein), beta1	−1.08 (0.63, 1.23)	0.489
DLG1	Disks, large homolog 1 (Drosophila)	1.07 (0.76, 1.38)	0.518
FOXP3	Forkhead box p3	1.19 (0.61, 1.77)	0.509
HAAO	Kydroxyanthranilic acid oxygenase (3‐hydroxyanthranilate 3,4‐dioxygenase)	1.09 (0.74, 1.44)	0.536
HNMT	Histamine n‐methyltransferase	1.04 (0.76, 1.32)	0.924
HRH1	Histamine receptor 1	1.18 (0.88, 1.48)	0.124
HRH2	Histamine receptor 2	1.16 (0.71, 1.60)	0.398
IDO1	Indoleamine 2,3‐dioxygenase	1.12 (0.61, 1.62)	0.604
IDO2	indoleamine 2,3‐dioxygenase 2	1.14 (0.34, 1.94)	0.559
IL10	Interleukin‐10	1.62 (0.40, 2.84)	0.266
IL13	Interleukin‐13	−1.17 (0.50, 1.20)	0.300
IL1B	Interleukin‐1beta	1.24 (0.45, 2.02)	0.350
IL2RA	(CD25) Interleukin‐2 receptor subunit alpha	1.77 (0.36, 3.19)	0.222
IL6	Interleukin‐6	1.07 (0.33, 1.81)	0.890
IL8	Interleukin‐8	1.15 (0.53, 1.76)	0.548
IL4	Interleukin‐4	−1.28 (0.49, 1.07)	0.131
IL17A	Interleukin‐17A	−1.10 (0.55, 1.27)	0.507
IL15	Interleukin‐15	−1.11 (0.53, 1.28)	0.536
INADL	InaD‐like (Drosophila)	1.06 (0.82, 1.31)	0.536
CCBL2	Kynurenine aminotransferase 3 (alias KAT3)	−1.05 (0.76, 1.14)	0.540
AADAT	Kynurenine aminotransferase 2 (alias KAT2)	−1.06 (0.60, 1.29)	0.973
GOT2	Kynurenine aminotransferase 4 (alias KAT4)	1 (0.83, 1.18)	0.851
KITLG	Kit‐ligand, Stem cell factor	−1.09 (0.61, 1.23)	0.644
KMO	Kynurenine 3‐monooxygenase	1.28 (0.56, 2.00)	0.574
KYNU	Kyureninase	1.16 (0.65, 1.68)	0.533
MAGI1	Membrane‐associated guanylate kinase, WW, and PDZ domain containing 1	1.08 (0.84, 1.31)	0.491
MPP5	Membrane protein, palmitoylated 5 (MAGUK p55 subfamily member 5)	1.03 (0.77, 1.29)	0.691
MPP7	Membrane protein, palmitoylated 7 (MAGUK p55 subfamily member 7)	1.04 (0.76, 1.33)	0.724
PPP1CB	Protein phosphatase 1, catalytic subunit, beta isozyme	1.01 (0.84, 1.19)	0.947
PPP2R5C	Protein phosphatase 2, regulatory subunit Beta	−1 (0.83, 1.17)	0.931
PRG2	Major basic protein	1.06 (0.74, 1.38)	0.968
PVRL3	Poliovirus signaling‐related 3	1.01 (0.85, 1.17)	0.838
QPRT	Quinolinic acid phosphoribosyltransferase	1.68 (0.04, 3.32)	0.612
TDO2	Tryptophan 2,3‐dioxygenase	1 (0.72, 1.28)	0.846
TGFB1	Transforming growth factor beta	1.05 (0.79, 1.30)	0.669
TJP2	zona occludens 2	1.06 (0.86, 1.25)	0.539
TJP3	zona occludens 3	1.1 (0.90, 1.30)	0.265
TLR1	Toll‐like receptor 1	1.04 (0.77, 1.32)	0.717
TLR2	Toll‐like receptor 2	1.05 (0.71, 1.38)	0.952
TLR3	Toll‐like receptor 3	−1.09 (0.56, 1.27)	0.649
TLR4	Toll‐like receptor 4	1.08 (0.82, 1.35)	0.550
TLR5	Toll‐like receptor 5	1.03 (0.70, 1.36)	0.806
TLR6	Toll‐like receptor 6	1.07 (0.79, 1.34)	0.761
TLR7	Toll‐like receptor 7	1.07 (0.74, 1.39)	0.581
TLR8	Toll‐like receptor 8	1.46 (0.47, 2.45)	0.363
TLR9	Toll‐like receptor 9	1.14 (0.42, 1.86)	0.390
TNF	Tumor Necrosis Factor – alpha (TNF‐a)	1.05 (0.73, 1.37)	0.706
TNFSF14	LIGHT/Tumor necrosis factor superfamily 14	1.1 (0.76, 1.44)	0.341
TPH1	Tryptophan hydroxylase 1	−1.02 (0.73, 1.22)	0.718
TPSAB1	Tryptase	1.03 (0.62, 1.43)	0.857
TPSB2	tryptase beta 2 (gene/pseudogene)	1.21 (0.79, 1.63)	0.389
AHR	Aryl Hydrocarbon Receptor	−1.04 (0.78, 1.15)	0.592
SOS1	Son of sevenless homolog 1 (Drosophila)	1.02 (0.84, 1.21)	0.863
MAPKAPK5	Mitogen‐activated protein kinase‐activated protein kinase 5	−1.08 (0.72, 1.13)	0.432
MKNK2	MAP kinase interacting serine/threonine kinase 2	−1.11 (0.67, 1.13)	0.433
B2M	Beta‐2 microglobulin	1.01 (0.79, 1.24)	0.987
ACTB	Actin, beta (house‐keeping gene [HKG])	1.02 (0.95, 1.10)	0.538
GAPDH	Glyceraldehyde‐3‐phosphate dehydrogenase	−1.02 (0.90, 1.05)	0.575
HGDC	Human Genomic DNA Contamination Control	−1.02 (0.60, 1.35)	0.658

#### Bile acid homeostasis

Serum FGF‐19 (114.6–113.5, *P* = 0.923) and 7*α* C4 (33.7–31.4, *P* = 0.292) were not significantly altered from baseline after 8 weeks of SBI therapy (Table 3).

We assessed the effect of SBI in four patients who had baseline fasting serum C4 > 49.1 ng/mL. There was a reduction in fasting serum C4 excretion (68.5 ± 25.8 [SD] at baseline compared to 50.5 ± 29.3 on SBI, *P* = 0.011). Similarly, the effect of SBI in four patients who had baseline fasting serum FGF‐19 < 79 pg/mL showed an increase in fasting serum FGF‐19 (55.4 ± 10.0 [SD] at baseline compared to 73.0 ± 8.6 on SBI, *P* = 0.057).

#### Microbiome analysis of duodenal brushings and stool

**Figure 3:**
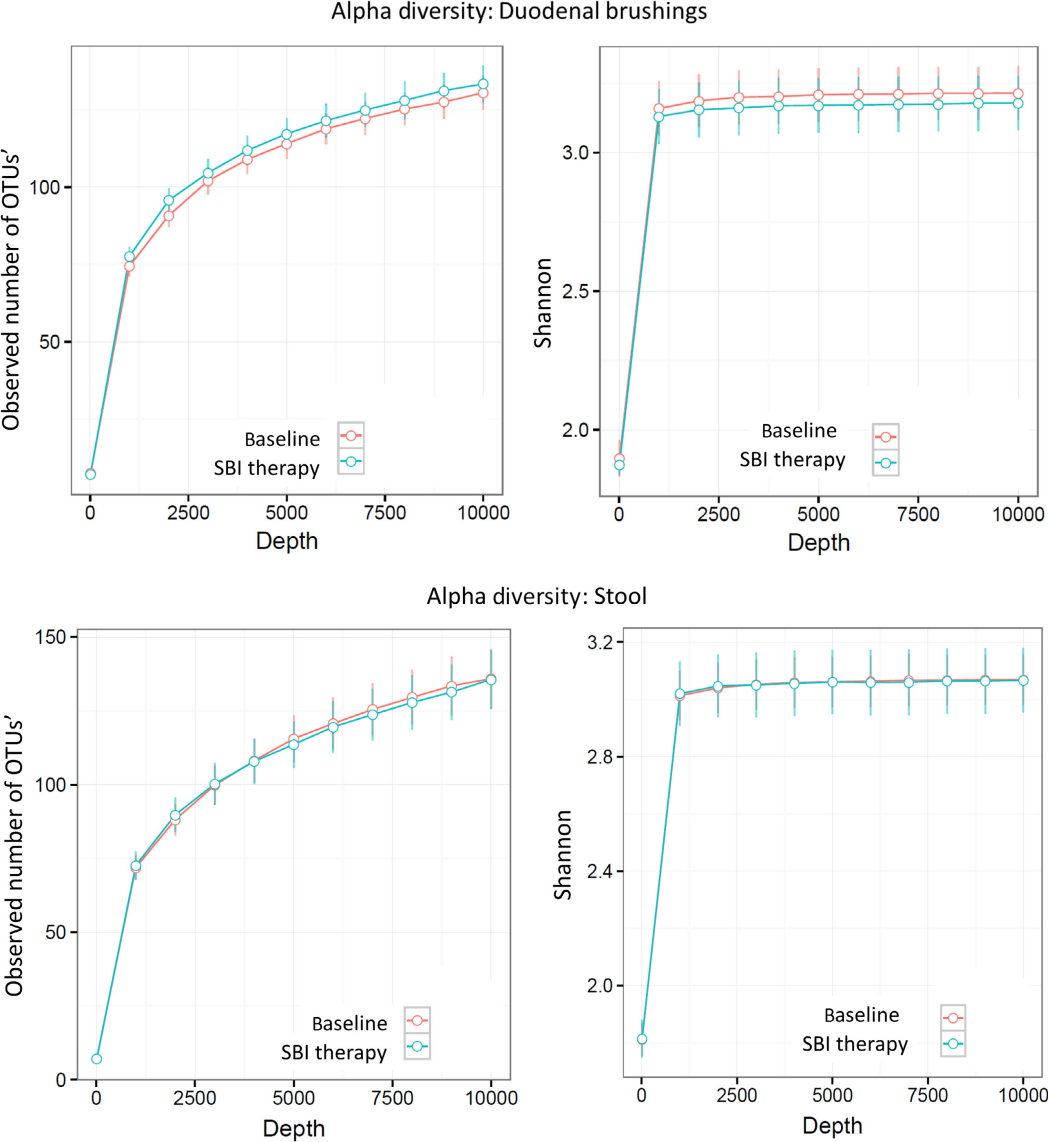
*α* diversity analysis of duodenal brushings (upper panel) and stool (lower panel) at baseline and during SBI therapy showing no significant differences.

**Figure 4:**
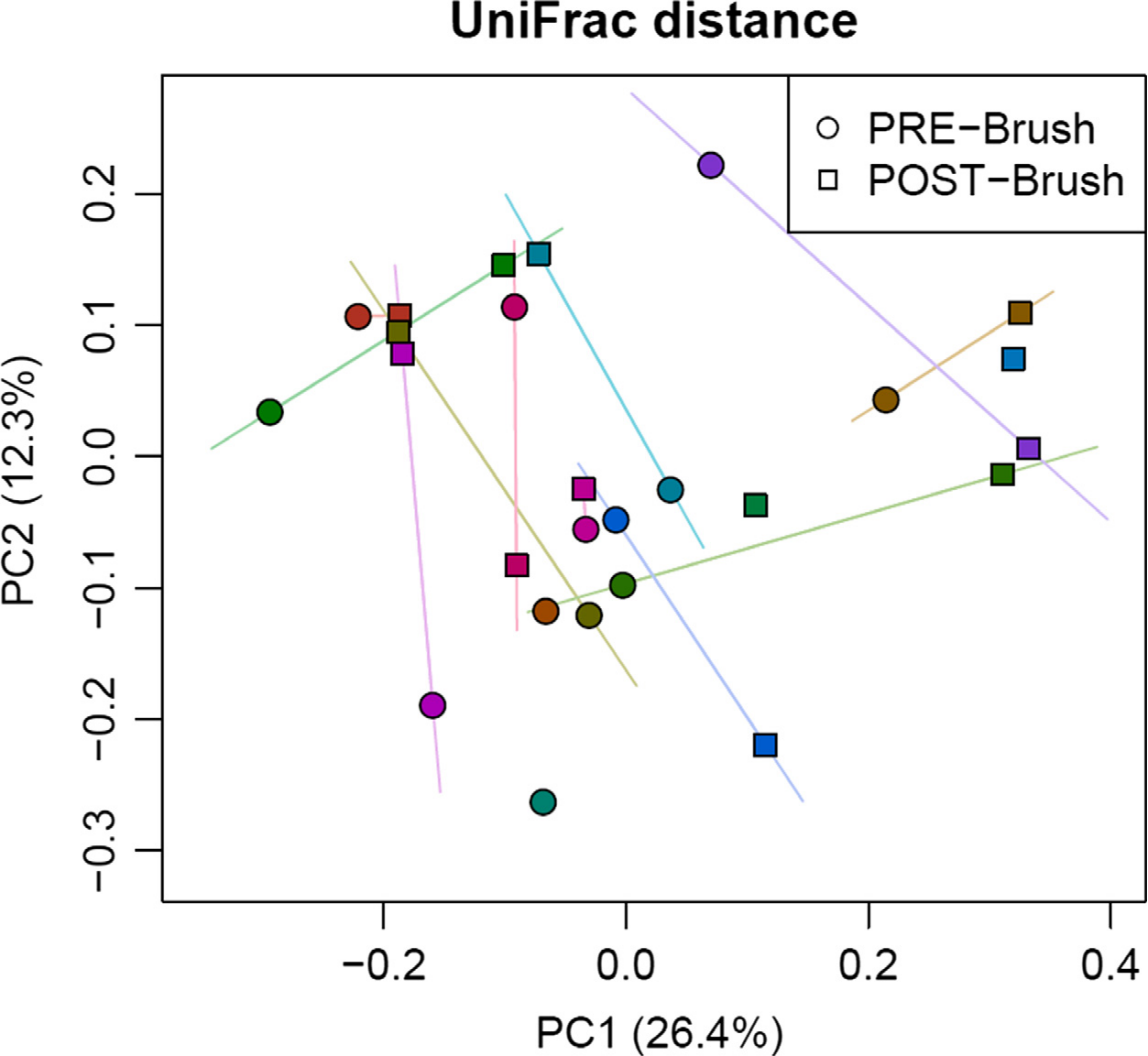
UniFrac distance‐based *β* diversity analysis of duodenal brushings showing changes during SBI therapy. Samples from the same subject are connected by straight lines. PRE‐BRUSH refers to microbial *β* diversity in the duodenal brushings prior to SBI therapy; POST‐BRUSH refers to the duodenal brushings following SBI therapy. UniFrac distance refers to the relative relatedness of community members by incorporating phylogenetic distances between observed organisms in the computation. The axes reflect principal components (PC) which are calculated linear combinations of the original variables that account for as much of the variance of the original data as possible.

**Figure 5:**
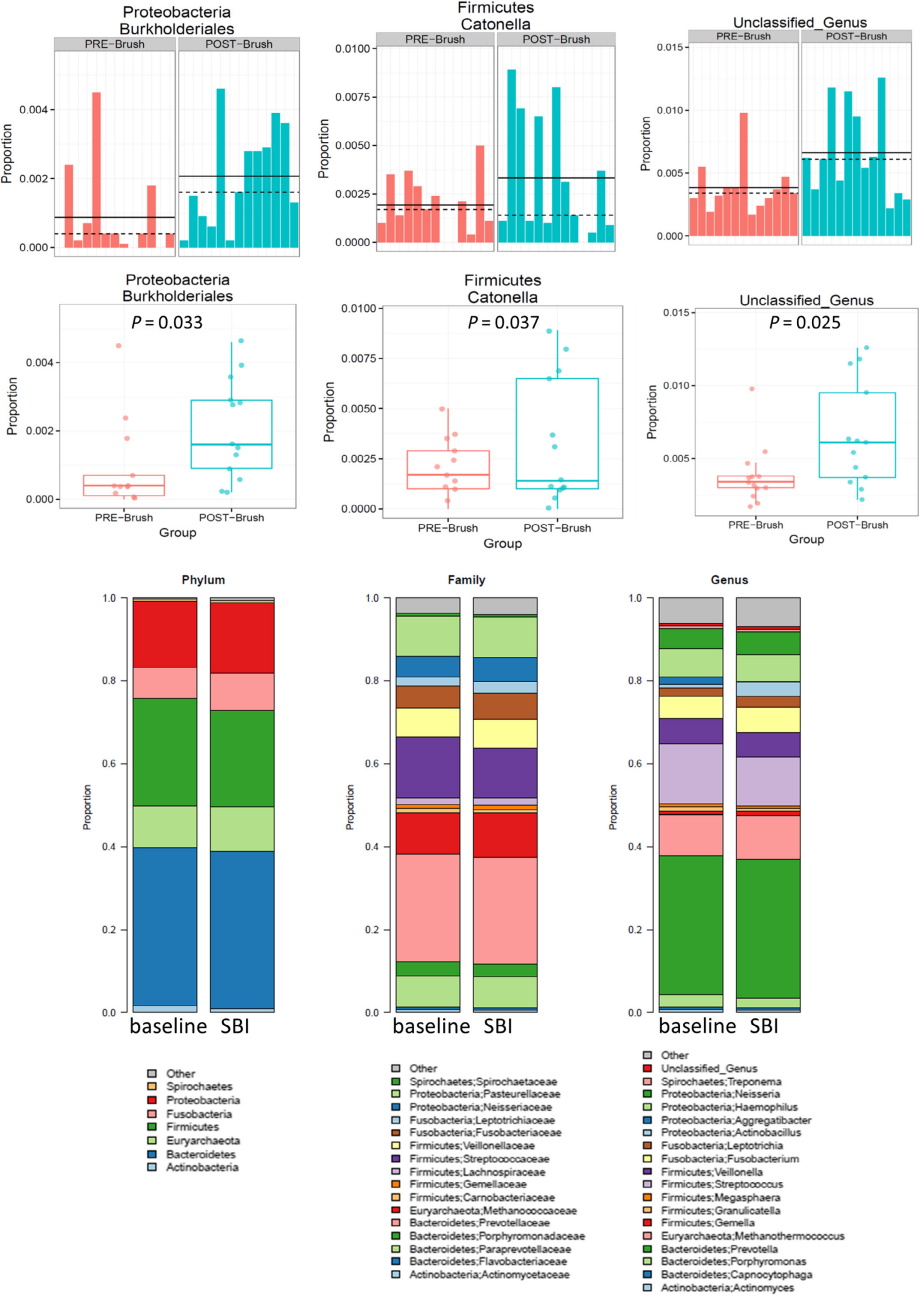
Taxonomic analysis of microbiome from duodenal brushings at baseline and with SBI therapy. UPPER PLOT: Abundance differences between baseline (PRE‐brush) and post‐SBI treatment (POST‐brush) in *Proteobacteria burkholderiales*,* Firmicutes catonella*, and unclassified genus bacteria. Individual patient data show three patients had post‐SBI treatment increases in all three genuses, but other individuals had either increases in one or two genuses. LOWER PLOT: No significant differences in other microbial flora based on taxonomic analysis of phylum, family or genus, or individual microbes.

**Figure 6:**
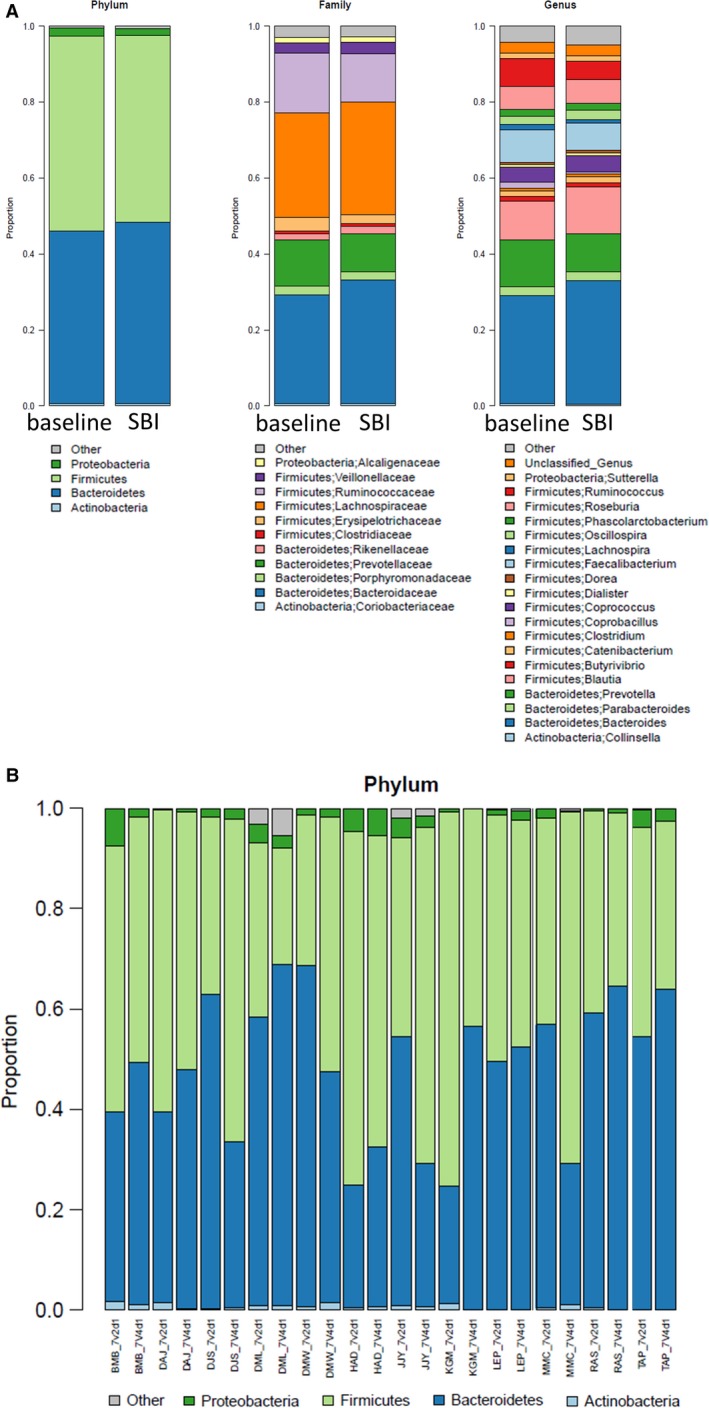
(A) Stool microbiome pre‐ and post‐SBI therapy (phylum, family, and genus) for 12 patients with samples available at baseline and on treatment. (B) Predominant organisms by phylum in stool pre‐ and post‐SBI therapy; V2 refers to visit 2 when sample was collected at baseline and V4 refers to visit 4 when sample was collected in the final days of treatment.

There were no significant effects of SBI therapy on *α* diversity of the microbiome in duodenal brushings or stool. Figure 3 shows rarefaction curves demonstrating species richness (observed number of OTUs) and evenness (Shannon index) for stool and duodenal brushings at baseline and on SBI therapy. However, the duodenal brushings microbiome showed considerable structure change as indicated by (unweighted) UniFrac analysis (Fig. 4) (*P* = 0.072). Taxonomic analysis revealed increases in the abundance of *Proteobacteria Burkholderiales, Fermicutes Catonella,* and unclassified genus organisms in duodenal brushings (Fig. 5) with SBI therapy. These increases were univariately significant (unadjusted *P* < 0.05); however, with correction for multiple testing using FDR control, the Q values for all these changes were >0.05. There were no significant differences in stool microbiome pre and posttherapy with SBI (Fig. 6).

## Discussion

Our single‐arm, open‐label study in 15 patients with IBS‐D evaluated the safety and effectiveness of SBI therapy (5 g, BID, for 8 weeks) on GI symptoms, epithelial barrier function, microbiome, and mucosal expression of pivotal genes in small bowel mucosa. The results of this study showed that SBI therapy for 8 weeks improved GI symptoms (i.e., number of stools per day, ease of passage, and sense of evacuation). These findings are consistent with previous studies which have shown that treatment with SBI improves GI symptoms in patients with IBS‐D, although that pilot study showed within‐group comparisons with benefit for some symptoms compared to baseline, rather than significant benefit compared to placebo treatment during the controlled trial (Wilson et al. 2013). Given the open‐label design of the trial, the clinical benefits require study in a placebo‐controlled trial. However, the present clinical study is the first to explore the mechanistic basis for any benefits observed in this patient population.

The pathogenesis of IBS is thought to be multifactorial with several complex interactions between various biological pathways (e.g., epithelial barrier function, genetic predisposition, low‐grade inflammation, change in small bowel and colonic flora) that culminate in alterations in intestinal homeostasis, bowel motility and sensitivity, and the manifestations of the wide spectrum of symptoms of IBS‐D. Traditionally, therapy has been aimed at resolving symptoms, but, as the pathophysiology of this disease becomes clearer, approaches directed at multimodal therapeutic targets are being proposed.

Increased intestinal permeability is considered an early event in IBS that leads to low‐grade immune cell infiltration of the gut mucosa (Bischoff et al. 2014). Evidence for the presence of such structural and functional disruptions in IBS has been provided by electron microscopy, which has detected enlarged spaces between epithelial cells and cytoskeletal condensation in gut biopsies of patients with IBS‐D (Martínez et al. 2013). Our study compared intestinal permeability at baseline and after SBI therapy for 8 weeks and found no significant change to suggest improvement of intestinal permeability. Although no clear mechanistic evidence of SBI mitigating altered gut barrier permeability was found, more detailed analyses using techniques such as electron microscopy are required to assess the histologic changes that might occur after therapy with SBI in IBS‐D patients. Indeed, future studies should appraise the effects of SBI therapy in patients with evidence of increased intestinal permeability or immune activation (increased inflammatory cells in the lamina propria) at baseline.

The relevance of the kynurenine pathway in IBS has been speculated for some time and, while it has been proposed that patients with IBS have a greater serum K:T ratio (due to enzyme mediated conversion of tryptophan to kynurenine) compared to controls (Clarke et al. 2009), a careful analysis showed elevated ratios in only 2 of the 10 male IBS patients studied. More convincing differences in the serum K:T ratio have been reported in patients with early HIV infection (Jenabian et al. 2015) and in patients with celiac disease (Torres et al. 2007). In the latter study, immunohistochemistry studies of intestinal biopsies showed an increased expression of the enzyme, indoleamine 2,3‐dioxygenase, interferon‐gamma, interleukin‐10 and transforming growth factor‐*β*, suggesting that a mechanism(s) dependent on tryptophan catabolism might regulate the immune responses in celiac disease. SBI therapy for 8 weeks did not significantly alter the K:T ratio in our study. Interestingly though, there was a measurable reduction in tryptophan levels after SBI therapy. One may have predicted that improvement of symptoms would more likely be associated with decreased levels of serotonin (5‐hydroxytryptamine) and, hence, higher levels of its precursors, 5‐hydroxytryptophan and tryptophan. However, alterations in peripheral tryptophan levels do not necessarily correlate with serotonergic function and could be explained by other factors, such as gender, in view of our predominantly female participants, since it is known that tryptophan levels vary significantly over the course of the menstrual cycle (Carretti et al. 2005).

Previous studies have identified genes linked to relevant pathways in IBS‐D pathogenesis and changes in fold expression of several genes relative to controls. Our analysis of 90 candidate genes related to inflammation, immune function, and tight junction regulation found no significant change in expression in small intestinal mucosa after therapy with SBI. There were borderline alterations in expression of mRNA of CLDN4 and IL4 genes, which are directly related to tight junction proteins and immune function, respectively. Although no significant changes were observed when comparing changes from baseline among IBS‐D subjects, our lab has shown in a separate pilot study that, when compared to controls (with normal duodenal biopsies on histopathology), there is upregulation of genes related to ion transport (INADL and MAGI1), immune function (TLR3 and IL15), and epithelial response to damage (PPP2R5C) (Camilleri et al. 2016a). These findings serve to support the importance of evaluating changes in gene expression of the small bowel mucosa to further elucidate mechanisms in the pathogenesis of this disease, the interactions between different biological pathways, the impact on intestinal homeostasis and the evaluation of the effects of therapy.

It is known that some patients with IBS‐D have increased levels of total fecal bile acids in the stool, which in turn results in increased intestinal water and electrolyte secretion, colonic transit and diarrhea (Valentin et al. 2016). Rarely, immunodeficiency has been associated with bile acid malabsorption (Dawson et al. 1979). SBI therapy does not seem to have a significant impact on bile acid homeostasis in patients with IBS‐D.

Our studies of the microbiome suggest that SBI therapy was associated with numerical changes in *Proteobacteria Burkholderiales* and *Firmicutes Catonella* in duodenal brushings, but there were no changes in stool microbiome. These observations suggest a hypothesis that the small intestinal mucosal microbiome may be altered by the administration of SBI therapy. *Proteobacteria Burkholderiales* is known to have the potential to catabolize a broad diversity of aromatic compounds (Pérez‐Pantoja et al. 2012). The biological significance of these changes in the duodenal mucosal microbiome will require further study, particularly, to assess the potential effects on barrier function and immune activation, especially in patients with IBS who already have baseline immune activation or increased expression of proteases or microRNAs that may predispose to alterations in functions, such as immune activation and visceral hypersensitivity (as reviewed in ref. Camilleri et al. 2016b). It is important to note that SBI is an IgG immunoglobin, which can certainly be digested in the small intestine before reaching the colon. This may certainly explain the lack of effects on stool microbiome or colonic permeability. This consideration led us to include the measurements of mRNA expression in small bowel biopsies, microbiome studies of duodenal brushings and small intestinal permeability (^13^C mannitol excretion during the first 2 h after ingestion).

### Limitations

Our study lacked a control treatment arm, and therefore, this is a limitation in the appraisal of the effects on symptoms; hence, we are not able to make generalized conclusions as to the specific effects of SBI therapy on the natural course of disease of IBS‐D, even in the presence of symptom improvement. However, it is important to note that the objective of our study was to expand on previous studies and to appraise the potential mechanisms for prior benefits observed with SBI relative to baseline symptoms (Wilson et al. 2013) by evaluating relevant biological markers and mRNA expression to appraise the impact of SBI therapy on relevant mechanisms involved in the pathogenesis of IBS‐D. Compliance with therapy was certainly adequate and there were no serious adverse events among the patients who completed the study; one patient withdrew from the study before completing all study procedures. Sample size was limited and based on a priori effect size calculations on a potentially achievable change in small bowel permeability (44% change from baseline on SBI therapy). The limited sample size may have impacted the study power to detect differences in the OTU levels between microbial species, leading to the negative findings regarding *α* diversity of the microbiome in the duodenal brushings. Another potential pitfall is that we studied stool microbiome, rather than microbiome of colonic mucosal biopsies or brushings, as we had tested in the duodenum. On the other hand, there were numerical (borderline significant) differences in duodenal microbiome with SBI therapy in *β* diversity and on taxonomic analysis which showed increases in microbial species, specifically *Proteobacteria Burkholderiales, Firmicutes Catonella,* and unclassified genus organisms.

The small sample size precluded a robust comparison of patients with normal or abnormal data at baseline. However, using the upper limit of normal for 0–2 h ^13^C‐mannitol excretion (Grover et al. 2016) of 0.228 (proportion of administered dose), we had six patients with high 0–2 h excretion at baseline, suggestive of increased small bowel permeability. These patients also did not have a significant reduction in small intestinal permeability.

Conversely, there were four patients with abnormal fasting serum C4 and four patients with abnormal fasting FGF‐19, and significant or borderline differences in these measurements were observed with SBI administration. Further studies in larger samples of patients are required to appraise effects on bile acid pathways. Overall, these observations reflect the concept that IBS‐D represents symptoms that may result from different pathophysiological mechanisms (Camilleri 2012). However, our observations provide useful information to propose mechanistic studies in subgroups, as well as providing the coefficient of variation to develop well‐powered studies, based on the different endpoints.

## Conclusion

We conclude from this open‐label study that treatment with SBI for 8 weeks may be beneficial in patients with IBS‐D, but there were no significant changes in intestinal permeability, gene expression, K:T ratio, or bile acid synthesis. The mechanism of benefit is unclear. There are intriguing numerical changes to suggest that the small intestinal microbiome is altered after therapy with SBI and this may play a role in improving epithelial barrier function and modulating immune activation. Importantly, the sample size in this study had sufficient power to detect realistic percent changes in the measures of small intestinal permeability and bile acid synthesis (through urine 0–2 h ^13^C‐mannitol excretion and fasting serum C4 levels, respectively). Larger studies are needed to further elucidate the role of SBI in the management of IBS‐D, particularly in patients with evidence of immune activation and barrier dysfunction at baseline. This study has estimated the coefficients of variation in diverse potential mechanisms in order to facilitate planning (e.g., sample size determination) for future randomized, controlled trials of SBI therapy in patients with IBS‐D.

## Conflict of Interest

No conflicts of interest.
